# The Association between Noncommercial Partnerships and Risk of HIV among Female Sex Workers: Evidences from a Cross-Sectional Behavioral and Biological Survey in Southern India

**DOI:** 10.1155/2013/108630

**Published:** 2013-03-04

**Authors:** Renuka Pulikallu Somanath, Ram Manohar Mishra, Niranjan Saggurti, Prabhakar Parimi

**Affiliations:** ^1^India HIV/AIDS Alliance, Hyderabad 500063, Andhra Pradesh, India; ^2^HIV & AIDS Program, Population Council, New Delhi 110003, India

## Abstract

This paper examines the association between female sex workers' (FSWs) noncommercial partnerships with risk of HIV in Andhra Pradesh, India. Data were drawn from a cross-sectional behavioral and biological survey conducted in 2009 among 3225 FSWs from Andhra Pradesh. Participants were asked about their sexual partnerships, condom use, and vulnerability factors and tested for HIV and sexually transmitted infections. The key independent variables considered were presence of a noncommercial sexual partner (no, yes) and the nature of such partnerships (regular, nonregular). FSWs who reported husband as noncommercial partner were considered to have a regular partner, while the rest were defined as having nonregular partners. Adjusted odds ratios (OR) and 95% confidence intervals (CI) were estimated to measure the associations between variables of interest. Almost three-fourths (74.8%) of FSWs reported having noncommercial partners (regular: 55.6%; nonregular: 19.3%). FSWs in nonregular partnerships were more likely to be HIV positive (13.1% versus 10.9%, adjusted OR: 1.4, 95% CI: 1.1–1.8), have syphilis (10.3% versus 4.2%, adjusted OR: 2.3, 95% CI: 1.6–3.3), use condoms inconsistently with occasional clients (21.0% versus 16.5%, adjusted OR: 1.5, 95% CI: 1.2–1.9), and report forced sex (25.1% versus 14.1%, adjusted OR: 1.9, 95% CI: 1.5–2.4) as compared to those in regular partnerships. HIV prevention programs need to emphasize safe sex behaviors, particularly among FSWs who have nonregular partners.

## 1. Introduction 

Currently, there are about 2.4 million people living with HIV in India [1]. The HIV epidemic in the country is predominantly heterosexual and is assumed to be fuelled mainly through unprotected sex with female sex workers (FSWs) [2]. Although the country has witnessed a declining trend in the adult HIV prevalence rate, the HIV prevalence is substantially high among most at risk groups such as FSWs [1]. Indeed, HIV prevalence among FSWs in the country is about 15 times higher than in the low-risk general population [3, 4]. Hence, HIV prevention programs in India continue to focus on the prevention of new HIV infections among FSWs and their sexual partners by promoting consistent condom use and treatment of sexually transmitted infections (STIs) in these groups [5].

While programmatic efforts across the country are believed to have resulted in higher levels of consistent condom use among FSWs with commercial sex partners [6–8], the consistent condom use practice remains at a much lower level with noncommercial partners such as husbands, boyfriends, pimps, and the police [9–11]. Due to low condom use, noncommercial partners have increasingly been recognized as a bridge group for targeted HIV prevention programs in India [9]; however, the effectiveness of interventions in increasing consistent condom use among noncommercial partners is not clear [9, 10, 12, 13]. 

The role of noncommercial partnerships in determining FSWs' vulnerability to HIV may be more complex and multidimensional than merely low condom use within such partnerships. For example, in a recent study, FSWs in southern India attributed their initiation of sex work and practice of high-risk behaviors (e.g., anal sex, inconsistent condom use in commercial sex) to increase financial debt being burdened by their noncommercial partners [14]. At the same time, many FSWs associated the departure of or abandonment by their husband to their economic hardship and subsequently to their initiation of sex work and practice of high risk behaviors [14]. Noncommercial sexual partners such as husbands, boyfriends, the police, and pimps were reported to act as protectors, as well as perpetrators of violence against FSWs in India [15, 16].

Studies have also shown that spousal sex has a positive effect on condom use in commercial sex among other high-risk population groups such as truckers. It has been noticed that married truckers were more likely to use condoms with nonmarital nonregular partners than their unmarried counterparts, possibly to protect themselves and their spouse from infection [17, 18]. Moreover, noncommercial relationships, especially with the husband, may have a positive effect on FSWs' sexual behavior, as they may result in less dependency on sex work (and hence less exposure to HIV and STI risk), safe sexual practices in commercial sex (e.g., consistent condom use), and psychosocial and physical protection from exogenous factors such as psychological harassment and physical and sexual violence. Thus, although many studies globally have documented the role of noncommercial partnerships in increasing FSWs' vulnerability to HIV mainly due to the low level of condom use within such relationships [9, 19–23], the differential effect of having husband or nonhusband as noncommercial partners received less attention in the literature, particularly in India. In this context, this study aims to examine the association between FSWs' noncommercial partnerships and their vulnerability to HIV in Andhra Pradesh, a southern state in India, with the highest burden of HIV in the country [1].

## 2. Materials and Methods

### 2.1. Data

We used data from a cross-sectional behavioral and biological survey among FSWs in eight districts of Andhra Pradesh. The survey districts were purposively selected based on the size of the FSWs population and sociocultural regions and included Chittoor, East Godavari, Guntur, Hyderabad, Karimnagar, Prakasam, Visakhapatnam, and Warangal districts. FSWs were defined as any female, 18 years or older who had sold sex in exchange for cash at least once in the one month preceding the survey. The target sample size per district was fixed at 400 completed interviews. Data collection was done between March and October 2009. 

A probability sampling method was used for selection of survey participants. Two sampling approaches were adopted following a comprehensive sampling frame development exercise spanning the entire district: (1) conventional cluster sampling was used for FSW practicing sex work at home, and in brothels, lodges, and *dabhas* (roadside eating establishments), where the population of FSWs was relatively stable; (2) conventional time-location cluster (TLC) sampling (dividing a site into several TLC and selecting the required number of TLCs randomly) was used for street-based FSWs. 

The survey collected behavioral information and biological specimens to test for STIs including HIV. Behavioral data, including information on demographics, sex work, sexual partners, and condom use, were collected through face-to-face interviews, using a pretested, precoded questionnaire translated into the local language, Telugu. Blood samples were collected for HIV and syphilis tests. Antecubital venipuncture blood sample (5 mL) was collected in a vacutainer, clotted for separation of serum, and stored at 2–8°C. A 30 mL urine sample was collected, from which 2 mL was stored in a urine specimen transport tube as per the protocol of M/s Gen-Probe Aptima Combo 2 Assay (Gen-Probe Incorporated, USA). Sera were tested for both HIV-1 and HIV-2 by Microlisa HIV kit (J. Mitra and Co. Pvt. Ltd, India, and GENEDIA HIV 1/2 ELISA 3.0 Kit, Gencross Life Science Corporation, Korea). Syphilis reactive serology was performed by Rapid Plasma Reagin Test Kit (Span Diagnostics Ltd, India) and was confirmed by Treponema Pallidum Hemagglutination Assay (TPHA) using Syphagen TPHA Kit. All cases with RPR reactive serology of any titre with TPHA positivity were considered positive. To diagnose *Neisseria gonorrhoeae* and *Chlamydia trachomatis*, urine samples were tested using Transcription-Mediated Amplification Assay and Dual Kinetic Assay (Gen-Probe Incorporated, USA).

The study was approved by all relevant institutional review boards (Health Ministry Screening Committee, Government of India, Scientific Advisory Committee of National AIDS Research Institute, Protection of Human Subjects Committee of Family Health International and Scientific Advisory Committee, and Ethical Committee of National Institute of Nutrition). A comprehensive informed consent process was developed that allowed respondents to become fully informed and have all questions answered before agreeing to participate in the survey. Respondents were allowed to consent to the behavioral portion of the survey and opt out of the biological portion, and, once the survey process began, they could discontinue at any time. Written consent was required before the interview process could begin. Other protective measures included oaths of confidentiality by all survey staff and the development of harm minimization guidelines and specimen and data safety guidelines. The opportunity to consult with a physician and receive an STI examination, syphilis results and treatment were the major benefits for individual respondents. The survey staff also provided referrals to STI clinics and integrated testing and counseling centers. Details of the survey methodology and preliminary survey findings are available elsewhere [6, 24, 25]. 

### 2.2. Measures

#### 2.2.1. Sociodemographic Characteristics

Sociodemographic characteristics included respondent's age (in completed years), formal schooling, which was defined as their ability to read and write (no, yes), marital status (never married, currently married, and divorced/separated), availability of any source of income other than sex work (no, yes), typology of sex work defined as primary place or mode of solicitation (home, public places, brothel, and phone), and duration of sex work (in completed years). Typology of sex work was derived based on the primary place for engaging in sex and mode of solicitation reported by FSWs. Duration of sex work was derived as the difference between FSWs' current age and the age when they initiated paid sex. 

#### 2.2.2. Presence and Nature of Noncommercial Partnerships

The two key independent variables in this study were presence of noncommercial sexual partners (no, yes) and nature of such partnerships (regular, nonregular). Presence of a noncommercial partner was measured by grouping FSWs into two categories: those who did not have a noncommercial partner and those who reported having at least one noncommercial partner at the time of survey. Commercial partners were either occasional clients (those whom FSWs did not know and recognize) or regular clients (those whom FSWs knew well and recognized). Noncommercial partners included regular partners (husband, boyfriend, and live-in partners) and nonregular partners (pimps, the police, local goon). We conceptualized nature of noncommercial partnerships (regular, nonregular) in the context of local norms and practices. It was realized that in most cases, spousal relationship may be considered to better represent regular relationships than other noncommercial partners such as boy friend or live-in partners. Hence, we defined the nature of noncommercial partnerships as regular, if FSWs reported their noncommercial regular partner as their husband (irrespective of their other sexual partnerships) and nonregular, if FSWs reported any noncommercial partner other than their husbands (e.g., boyfriend, live-in partner, pimps, the police, and local goon). 

#### 2.2.3. Outcome Indicators

The outcome indicators included in the study were inconsistent condom use with occasional and regular clients, inconsistent condom use with nonregular partners, HIV and STI status, experience of violence in the past six months (no, yes), experience of forced sex (no, yes), and practice of anal sex with commercial partners (no, yes). Inconsistent condom use was defined as failure to use a condom in every vaginal sexual encounter with a particular type of partner during the past one week. A respondent was considered HIV-positive if she tested positive for either HIV-1 or HIV-2 or both. Prevalence of the following three STIs was included syphilis, *Gonorrhoeae*, and *Chlamydia*. Experience of violence was defined as whether the FSW had been beaten by any individual in the past six months (no, yes). Experience of forced sex was defined as whether the FSW had been physically forced to have sexual intercourse with any individual in the past one year (no, yes). Practice of anal sex was defined as having anal sex with any commercial partner in the past one week (no, yes). Inconsistent condom use in anal sex was measured by failure to use a condom at last anal sex, as information on use of condoms in every anal sex encounter was not collected in the survey. 

To understand the possible mechanisms by which nature of noncommercial relationships could affect FSWs' vulnerability to HIV, we examined differences in sex work characteristics and perpetrators of violence among FSWs by nature of noncommercial partnerships. Sex work characteristics which we examined were the number of days the FSW had worked as a sex worker in the past one week (0–2, 3–5, 6, or more) and the number of commercial sexual partners she had entertained in the past one week (low (0–5), medium (6–9), and high (10 or more)). The categorization of number of commercial sexual partners was based on the grouping adopted by India's National AIDS Control Program [2]. The information on perpetrators of violence (strangers, madams, other FSWs, paying partners, nonpaying partners, and police/pimps) was elicited among those who reported to have experienced violence in the past six months using a multiple response question. 

### 2.3. Statistical Analyses

Cross tabulations were made to examine the differences between sociodemographic characteristics, sexual behavior, and STI/HIV prevalence among FSWs by presence and nature of noncommercial partnerships. Chi-square and unpaired *t*-tests were applied to test significance of the differences in proportions and differences in mean values, respectively. Crude odds ratios (crude OR) and adjusted odds ratios (adjusted OR) along with corresponding 95% confidence intervals (CI) were estimated to measure the effects of the presence of noncommercial partnerships and nature of such partnerships on FSWs' vulnerability to HIV and their HIV/STI status. This was done by estimating separate logistic regression models with the following binary (no, yes) outcome variables: (1) whether tested positive for HIV; (2) whether tested positive for syphilis, *Gonorrhoeae*, and *Chlamydia *(separate models for each); (3) whether used condoms inconsistently with occasional clients; (4) whether used condom inconsistently with regular clients; (5) whether experienced physical violence in the past six months; (6) whether experienced forced sex in the past 12 months; (7) whether ever had anal sex; (8) whether practiced anal sex in the past seven days; and (9) nonuse of condom at last anal sex. The crude OR were estimated by keeping only one of the two key independent variables (i.e., presence of any noncommercial partner, nature of noncommercial partnerships) as an explanatory variable in the logistic regression model, whereas the adjusted OR was computed by controlling for the effects of sociodemographic characteristics described previously. 

## 3. Results

### 3.1. Sociodemographic Characteristics

Of 3225 FSWs who participated in the survey, 2415 (74.8%) reported having a noncommercial partner (regular: 1792 (55.6%), nonregular: 623 (19.3%)) (Table 1). Compared to FSWs who did not have a noncommercial partner, a larger proportion of FSWs with noncommercial partners were younger (mean age: 30.9 years versus 29.8 years, *P* = 0.012) and had been working as sex worker for a shorter period (mean duration: 6.6 years versus 5.2 years, *P* < 0.001). A higher proportion of FSWs who did not have a noncommercial partner, compared to those who reported having a noncommercial partner, were not currently married (never married: 23.9% versus 4.3%, *P* < 0.001; divorced/separated: 55.4% versus 11.1%, *P* < 0.001) and had no source of income other than sex work (62.2% versus 48.2%, *P* < 0.001). Similarly, among FSWs who had noncommercial partners, a larger proportion of those in unsteady relationships had no source of income other than sex work (60.6% versus 43.9%, *P* < 0.001) compared to those who reported being in a steady relationship. 

### 3.2. Prevalence of HIV, STI, Risk Behaviors, and Vulnerability Factors by Presence of Noncommercial Partnerships

HIV prevalence was found to be higher among FSWs who did not have noncommercial partners compared to those who reported a noncommercial partner (18.3% versus 11.1%, adjusted OR: 0.5, 95% CI: 0.4–0.7) (Table 2). However, FSWs who had a noncommercial partner were more likely than others to have experienced physical violence in the past six months (25.9% versus 18.9%, adjusted OR: 1.7, 95% CI: 1.4–2.1), experienced forced sex in past 12 months (16.9% versus 13.4%, adjusted OR: 1.4, 95% CI: 1.1–1.7), and practiced anal sex in the past one week (20.9% versus 12.5%, adjusted OR: 1.7, 95% CI: 1.4–2.1).

### 3.3. Prevalence of HIV, STI, Risk Behaviors, and Vulnerability Factors by Nature of Noncommercial Partnerships

FSWs who reported having nonregular partners, compared to those who had regular partners, were more likely to have HIV (13.1% versus 10.9%, adjusted OR: 1.4, 95% CI: 1.1–1.8), to have syphilis (10.3% versus 4.2%, adjusted OR: 2.3, 95% CI: 1.6–3.3), to practice inconsistent condom use with occasional clients (21.0% versus 16.5%, adjusted OR: 1.5, 95% CI: 1.2–1.9), to have experienced physical violence in the past six months (37.4% versus 21.9%, adjusted OR: 1.5, 95% CI: 1.2–1.9), to have experienced forced sex in the past 12 months (25.1% versus 14.1%, adjusted OR: 1.9, 95% CI: 1.5–2.4), and to have practiced anal sex in the past one month (30.1% versus 17.7%, adjusted OR: 1.9, 95% CI: 1.6–2.5) (Table 3).

### 3.4. Sex Work Characteristics and Perpetrator of Violence by Nature of Noncommercial Partnerships

FSWs in nonregular sexual partnerships, compared to those in regular sexual partnerships, had worked more days as sex workers in the past one week (mean number of days: 4.9 versus 4.1, *P* = 0.002) and had more commercial partners in past one week (mean number 13.9 versus 10.3, *P* < 0.001) (Table 4). A larger proportion of FSWs in unsteady noncommercial partnerships, compared to those who were steady relationships, reported experience of violence perpetrated by paying partners (37.7% versus 19.1%, *P* < 0.001) and police/pimps (15.9% versus 7.4%, *P* = 0.001).

## 4. Discussion

This cross-sectional study shows that despite relatively higher exposure to vulnerability factors (such as violence, forced sex, and anal sex) HIV prevalence was lower among FSWs with noncommercial partners compared to those who reported no noncommercial partners. Among the subgroup of FSWs who reported having noncommercial partners, those living with their husband were found to have lower HIV and syphilis prevalence, less exposure to vulnerability factors, and higher condom use in commercial sex encounters compared to those who reported having nonregular partnerships with partners such as boyfriends, live-in partners, pimps, the police, and local goon. 

The study finding that FSWs who only have commercial partners are most at risk for HIV and syphilis corroborates results of earlier studies that FSWs' commercial sexual partnerships contribute greatly to the HIV epidemic in India [26–28]. The higher HIV vulnerability of FSWs who were in nonregular partnerships compared to those who were in regular partnerships suggests that nature noncommercial relationships may have different effects on FSWs' vulnerability to HIV. Living with one's husband—who is a socially accepted steady noncommercial sexual partner—appears to safeguard FSWs from violence, forced sex, and risky sexual practices such as inconsistent condom use in commercial sex and anal sex. A possible mechanism by which living with one's husband may affect FSWs is by making FSWs less dependent on sex work for their income, as seen in the finding that FSWs living with their husband reported spending fewer days engaged in sex work and entertaining fewer commercial sex partners compared to other FSWs. Another possible positive effect of FSWs living with their husband could be the involvement of such FSWs in nonsex work-related economic activities (e.g., selling vegetables/fruits or working as daily laborer), to get greater autonomy and freedom for mobility. Even if FSWs get involved in any economic activity just for sake of keeping themselves “good” in the eyes of families and society, the additional income may also reduce their economic dependency on sex work.

Studies have documented that greater economic dependence on sex work increases FSWs' vulnerability to HIV [11, 14, 29]. In contrast, lesser dependence on sex work may allow FSWs greater flexibility and autonomy in negotiating for condom use with commercial partners as well as avoiding interaction with troublesome clients (e.g., those who are drunk). This was reflected, at least to some extent, by the smaller proportion of FSWs living with their husbands who experienced violence perpetrated by commercial sex partners and police/pimps compared to those who reported other men as noncommercial partners. While we recognize that low condom use in noncommercial partnerships including with husbands is an important concern for HIV prevention programs, this study indicates the possible positive health benefits of living with one's husband—an indicator of a regular noncommercial sexual partnership. 

The results of this study need to be interpreted with an understanding that adding more number of partners may apparently increase the probability of being exposed to vulnerability factors such as violence, forced sex, and anal sex. Hence, we found higher proportion of FSWs reporting such factors among those who had noncommercial partners compared to those who only had commercial partners. However, the key message of this study is that among the subgroup of FSWs who had noncommercial partners, living with one's husband may bring some desirable changes in FSWs' adoption of safe sex behaviors. It has been observed that in India, most often husbands are not aware of their spouse's involvement in sex work, while other noncommercial partners are often actively involved in the management of their partner's sex work [9, 30]. As a result, husbands are often not reached by HIV prevention programs [30]. Findings from this study suggest that HIV prevention programs should work with FSWs' having noncommercial partners such as those pimps, local goon, and the police in order to further reduce the “ping-pong” HIV transmission between them. 

Although the current research provides important insights to support better HIV prevention efforts for FSWs, the findings must be interpreted with caution. We considered spousal relationships to be a better indicator of regular sexual partnership, which implies the social, economic, psychological, and physical interdependences of both partners. Although there could be few cases where FSWs may find the previous characteristics in relationships with men other than their husband, such cases are likely to be too small to affect the conclusions of this study considering the local social and cultural context. Also, the HIV status of a FSW may affect her noncommercial relationships because those HIV positive FSWs are likely to be deserted by their noncommercial partners if her HIV status is known to them. Finally, the results are based on self-reports, and effects of social-desirability bias cannot be denied. 

## 5. Conclusions 

In conclusion, the study results show that although FSWs who only have commercial sex partnerships have the highest burden of HIV, vulnerability to HIV is significantly higher among those who have noncommercial partnerships, particularly those who are in nonregular sexual partnerships. HIV prevention efforts should continue to focus on FSWs' commercial sex partnerships; however, at the same time, FSWs' nonregular sexual partnerships should also be the focus of programs with equal intensity and importance. Finally, we do not intend to suggest that concerns regarding low consistent condom use with regular noncommercial partners are less important. Rather, we intend to highlight possible positive mechanisms associated with regular sexual relationships to less risk taking as compared to nonregular sexual relationships. It is important therefore to strengthen the HIV prevention interventions with FSWs in promoting safe sexual behaviors in all sexual relationships, including those in regular and nonregular relationships. 

## Figures and Tables

**Table 1:** Sociodemographic characteristics of female sex workers by presence and nature of noncommercial partnerships, Andhra Pradesh, 2009 (*N* = 3225).

Sociodemographic characteristics	Presence of noncommercial partners			Nature of noncommercial partnership		
	No	Yes	*P* value	Regular	Nonregular	*P* value
	(*N* = 810)	(*N* = 2415)		(*N* = 1792)	(*N* = 623)	
Age ≥30 years	58.3	49.7	<0.001	44.6	51.4	0.003
Mean age in years (SD)	30.9 (7.9)	29.8 (6.4)	0.012	29.9 (6.2)	29.6 (6.9)	0.250
Formal schooling	41.2	40.9	0.878	37.8	50.1	<0.001
Marital status						
Never married	23.9	4.3	<0.001	—	16.8	—
Currently married	20.8	84.6	<0.001	—	40.3	—
Divorced/separated	55.4	11.1	<0.001	—	42.9	—
Typology of sex work						
Home based	17.9	16.6	0.410	17.5	14.1	0.057
Public place based	64.6	65.2	0.737	64.9	66.0	0.647
Brothel based	8.1	9.0	0.383	7.9	12.1	0.002
Phone based	9.5	9.1	0.745	7.9	9.6	0.365
Mean duration of sex work (SD)	6.6 (3.7)	5.2 (4.6)	<0.001	4.9 (4.1)	6.1 (5.2)	0.002
No source of income other than sex work	62.2	48.2	<0.001	43.9	60.6	<0.001

SD: standard deviation.

*P* values are obtained by comparing values for FSWs with and without noncommercial partners. Significances of the differences in percentages were tested using *Z*-test. Significances of differences in average values were tested using unpaired *t*-test.

**Table 2:** Prevalence of HIV, sexually transmitted infections, risk behaviors, and vulnerability factors by presence of noncommercial partners among female sex workers, Andhra Pradesh, 2009 (*N* = 3225).

Prevalence of HIV, sexually transmitted infections, risk behaviors, and vulnerability factors	Presence of noncommercial partner			
	No	Yes	Crude OR	Adjusted OR
	(*N* = 810)	(*N* = 2415)	(95% CI)	(95% CI)
HIV	18.3	11.5	0.6 (0.5–0.7)	0.5 (0.4–0.7)
Syphilis	7.2	5.8	0.8 (0.6–1.1)	1.0 (0.7–1.4)
*Neisseria gonorrhoeae *	3.0	2.7	0.9 (0.5–1.4)	0.7 (0.5–1.2)
*Chlamydia trachomatis *	4.0	3.3	0.8 (0.6–1.3)	0.9 (0.6–1.7)
Inconsistent condom use with occasional clients	16.0	17.7	1.1 (0.9–1.4)	1.1 (0.9–1.5)
Inconsistent condom use with regular clients	16.9	17.8	1.1 (0.8–1.4)	1.1 (0.8–1.3)
Experience of physical violence, past 6 months	18.9	25.9	1.5 (1.2–1.8)	1.7 (1.4–2.1)
Experience of forced sex, past 12 months	13.4	16.9	1.3 (1.1–1.7)	1.4 (1.1–1.7)
Ever had anal sex	18.5	26.1	1.6 (1.3–1.9)	1.5 (1.2–1.9)
Practice of anal sex, past one week	12.5	20.9	1.9 (1.5–2.3)	1.7 (1.4–2.1)
No condom use in last anal sex	7.8	8.2	1.0 (0.4–2.1)	0.7 (0.4–1.5)

OR: odds ratio; CI: confidence interval.

FSWs who reported not having noncommercial partners were considered as reference category for computing crude and adjusted odds ratios.

Odds ratios were adjusted for FSWs' current age (in completed years), formal schooling (no, yes), typology of sex work (home, brothel/lodge, street, and phone), marital status (never married, currently married, and divorced/separated), source of income other sex work (no, yes), and duration of working as sex worker (in completed years).

**Table 3:** Prevalence of HIV, sexually transmitted infections, risk behaviors, and vulnerability factors by nature of noncommercial Partnerships, Andhra pradesh, 2009 (*N* = 2415).

Prevalence of HIV, sexually transmitted infections, risk behaviors, and vulnerability factors	Nature of noncommercial partnerships			
	Regular	Nonregular	Crude OR	Adjusted OR
	(*N* = 1792)	(*N* = 623)	(95% CI)	(95% CI)
HIV	10.9	13.1	1.2 (0.9–1.6)	1.4 (1.1–1.8)
Syphilis	4.2	10.3	2.6 (1.9–3.7)	2.3 (1.6–3.3)
*Neisseria gonorrhoeae *	2.3	2.8	0.8 (0.4–1.5)	0.9 (0.5–1.7)
*Chlamydia trachomatis *	4.3	3.0	1.5 (0.9–2.3)	1.3 (0.8–2.2)
Inconsistent condom use with occasional clients	16.5	21.0	1.3 (1.1–1.8)	1.5 (1.2–1.9)
Inconsistent condom use with regular clients	17.5	18.6	1.1 (0.8–1.4)	1.1 (0.9–1.5)
Experience of physical violence, past 6 months	21.9	37.4	2.1 (1.7–2.6)	1.9 (1.5–2.3)
Experience of forced sex, past 12 months	14.1	25.1	2.0 (1.6–2.6)	1.9 (1.5–2.4)
Ever had anal sex	22.2	35.2	1.9 (1.5–2.3)	1.9 (1.6–2.4)
Practice of anal sex, past one week	17.7	30.1	1.9 (1.6–2.5)	2.1 (1.7–2.6)
No condom use in last anal sex	8.4	8.0	0.9 (0.5–1.7)	1.1 (0.5–2.1)

OR: odds ratio; CI: confidence interval.

Analyses were restricted among FSWs who reported having any noncommercial partner.

FSWs with regular partners were considered as reference category for computing crude and adjusted odds ratios.

Odds ratios were adjusted for FSWs' current age (in completed years), formal schooling (no, yes), typology of sex work (home, brothel/lodge, street, and phone), marital status (never married, currently married, and divorced/separated), source of income other sex work (no, yes), and duration of working as sex worker (in completed years).

**Table 4:** Sex work characteristics and perpetrator of violence by nature of noncommercial partnerships among female sex workers, Andhra Pradesh, 2009.

Sex work characteristics and perpetrator of violence	Nature of noncommercial partnerships		
	Regular	Nonregular	*P* value
	(*N* = 1792)	(*N* = 623)	
Number of days worked as sex worker in past one week			
Up to 2	13.1	7.2	<0.001
3–5	68.4	56.1	<0.001
6 or more	18.6	36.7	<0.001
Mean number of days (SD)	4.1 (1.5)	4.9 (1.6)	0.002
Number of commercial partners in past one week			
Low (Up to 5)	18.2	9.0	<0.001
Medium (6–9)	32.8	26.3	0.003
High (10 or more)	49.0	64.7	<0.001
Mean number of clients (SD)	10.3 (6.3)	13.9 (9.0)	<0.001
Perpetrator of violence in past six months*	(*N* = 393)	(*N* = 233)	
Strangers	40.3	43.7	0.404
Madam	2.7	5.2	0.086
Other female sex workers	17.3	6.3	0.001
Commercial sex partners	19.1	37.7	<0.001
Nonpaying partners	25.6	20.5	0.147
Police/Pimps	7.4	15.9	0.001

SD: standard deviation.

Analyses were restricted among FSWs who reported having any noncommercial partner.

*Among FSWs who reported to have experienced violence in past six months.

*P* values are obtained by comparing values for FSWs with regular and nonregular noncommercial partners. Significances of the differences in percentages were tested using *Z*-test. Significances of differences in average values were tested using unpaired *t*-test.
